# Mass Casualty Incident Training in Immersive Virtual Reality: Quasi-Experimental Evaluation of Multimethod Performance Indicators

**DOI:** 10.2196/63241

**Published:** 2025-01-27

**Authors:** Anke Sabine Baetzner, Yannick Hill, Benjamin Roszipal, Solène Gerwann, Matthias Beutel, Tanja Birrenbach, Markus Karlseder, Stefan Mohr, Gabriel Alexander Salg, Helmut Schrom-Feiertag, Marie Ottilie Frenkel, Cornelia Wrzus

**Affiliations:** 1 Institute of Sports and Sports Sciences Heidelberg University Heidelberg Germany; 2 Department of Human Movement Sciences, Faculty of Behavioral and Movement Sciences Vrije Universiteit Amsterdam Amsterdam Netherlands; 3 Institute of Brain and Behaviour Amsterdam Amsterdam Netherlands; 4 Lyda Hill Institute for Human Resilience University of Colorado Colorado Springs Colorado Springs, CO United States; 5 MindConsole GmbH Graz Austria; 6 Department of Emergency Medicine Inselspital University Hospital, University of Bern Bern Switzerland; 7 Medical Faculty Heidelberg University Heidelberg Germany; 8 Department of Anesthesiology University Hospital Heidelberg Heidelberg Germany; 9 General-, Visceral- and Transplantation Surgery University Hospital Heidelberg Heidelberg Germany; 10 AIT Austrian Institute of Technology Vienna Austria; 11 Psychology in Health Care, Faculty Health, Safety, Society Furtwangen University Freiburg Germany; 12 Psychological Institute and Network Aging Research Heidelberg University Heidelberg Germany

**Keywords:** prehospital decision-making, disaster medicine, emergency medicine, mass casualty incident, medical education, eye tracking, emergency simulation, virtual reality

## Abstract

**Background:**

Immersive virtual reality (iVR) has emerged as a training method to prepare medical first responders (MFRs) for mass casualty incidents (MCIs) and disasters in a resource-efficient, flexible, and safe manner. However, systematic evaluations and validations of potential performance indicators for virtual MCI training are still lacking.

**Objective:**

This study aimed to investigate whether different performance indicators based on visual attention, triage performance, and information transmission can be effectively extended to MCI training in iVR by testing if they can discriminate between different levels of expertise. Furthermore, the study examined the extent to which such objective indicators correlate with subjective performance assessments.

**Methods:**

A total of 76 participants (mean age 25.54, SD 6.01 y; 45/76, 59% male) with different medical expertise (MFRs: paramedics and emergency physicians; non-MFRs: medical students, in-hospital nurses, and other physicians) participated in 5 virtual MCI scenarios of varying complexity in a randomized order. Tasks involved assessing the situation, triaging virtual patients, and transmitting relevant information to a control center. Performance indicators included eye-tracking–based visual attention, triage accuracy, triage speed, information transmission efficiency, and self-assessment of performance. Expertise was determined based on the occupational group (39/76, 51% MFRs vs 37/76, 49% non-MFRs) and a knowledge test with patient vignettes.

**Results:**

Triage accuracy (*d*=0.48), triage speed (*d*=0.42), and information transmission efficiency (*d*=1.13) differentiated significantly between MFRs and non-MFRs. In addition, higher triage accuracy was significantly associated with higher triage knowledge test scores (Spearman ρ=0.40). Visual attention was not significantly associated with expertise. Furthermore, subjective performance was not correlated with any other performance indicator.

**Conclusions:**

iVR-based MCI scenarios proved to be a valuable tool for assessing the performance of MFRs. The results suggest that iVR could be integrated into current MCI training curricula to provide frequent, objective, and potentially (partly) automated performance assessments in a controlled environment. In particular, performance indicators, such as triage accuracy, triage speed, and information transmission efficiency, capture multiple aspects of performance and are recommended for integration. While the examined visual attention indicators did not function as valid performance indicators in this study, future research could further explore visual attention in MCI training and examine other indicators, such as holistic gaze patterns. Overall, the results underscore the importance of integrating objective indicators to enhance trainers’ feedback and provide trainees with guidance on evaluating and reflecting on their own performance.

## Introduction

### Overview

Medical first responders (MFRs) are confronted with extreme demands at mass casualty incidents (MCIs), during which they must attend to more patients than their resources allow [1]. Whether those situations are due to natural disasters, accidents, or terrorist attacks, peak performance of MFRs under such demanding circumstances is crucial. However, current MCI training is insufficient to prepare MFRs adequately for MCIs due to low immersion and realism of classroom learning and scarce real-life exercises that are typically used [2-4].

An increasingly popular tool for MCI training is immersive virtual reality (iVR; [3,5]). iVR is typically experienced through a head-mounted display (HMD) that elicits the user’s impression of being completely surrounded by a 3D, virtual world [6-8]. In addition, users are often able to move around, explore, and interact with their environment (eg, by walking and using controllers). Especially in the context of MCI training, iVR offers the possibility to train numerous scenarios in a safe, flexible, and resource-efficient way [5]. iVR training applications also allow for high-quality assessments of performance, which are crucial for enabling systematic and structured training [9] and for assessing the degree to which MFRs are prepared for MCIs. Moreover, high-quality performance assessments allow for the evaluation of training effectiveness and the comparison of training methods, contributing to an ongoing improvement of MCI preparedness [10]. However, a systematic evaluation of performance indicators for iVR MCI training is still missing. This study aims to fill this gap by evaluating the usefulness of different performance indicators in virtual MCI scenarios. Specifically, we first provide an overview of the potential of iVR for MCI simulation training. Next, we discuss various performance indicators that cover different aspects of the performance of MFRs who arrive first at an MCI scene (ie, visual attention, triage accuracy, triage speed, and information transmission). Finally, we introduce self-rated performance as a potential tool for a holistic assessment.

### MCI Training and iVR

The gold standard for MCI training is to take part in real-life scenarios with patient actors and manikins [11]. However, such exercises are particularly resource-intensive in terms of preparation time and costs. Therefore, technological training solutions have been developed to increase the level of preparedness, such as training in iVR [5].

iVR provides near-realistic training sessions that require fewer personnel and financial resources compared to real-life MCI exercises with patient actors [4]. iVR also allows the creation of an infinite number of scenarios that can be quickly and flexibly adapted to training objectives [8]. By providing a safe training environment where mistakes can be made and scenarios can be repeated until they are handled correctly [12,13], iVR can be used in training for otherwise dangerous scenarios, such as fire or terrorist attacks. The training and its challenges can also be adapted to individual needs and expertise levels, thus providing an opportunity to maintain a balance by being complex enough to trigger learning without leading to overload [8,14]. On the basis of their current skills, MFRs may be placed in virtual scenarios with few patients and distractions or may train in large-scale MCIs with more patients under complex conditions (eg, nighttime and difficult terrain). Another advantage of iVR-based training is the ability to implement automated monitoring of objective performance indicators, which can help improve both the trainees’ learning process and their preparedness [15]. Furthermore, iVR facilitates the inclusion of novel performance indicators, such as visual attention assessed with eye-tracking, which integrates seamlessly with the technology [16].

### Performance Indicators

#### Overview

Performance indicators can be validated with the known-groups validation approach [17,18] which tests the expectation that distinct groups (eg, age groups, experts, and novices) differ on a certain measure [19]. Effective indicators should distinguish between experts and novices, as experts, by definition, possess the knowledge and skills to consistently perform at high levels [20]. Previous studies have classified experts and novices based on various measures, including having or not having a specific profession or certificate, often intertwined with (years of) job experience (eg, medical students vs physicians) [21]. In addition, experts generally possess more knowledge than novices [22].

Because performance assessments should capture multiple dimensions of performance [9], we evaluate three areas for a broad performance assessment of the first MFRs who arrive at an MCI: (1) the process of gaining an overview of the situation when arriving on scene, which could be assessed based on visual attention, (2) the triage process, and (3) the efficiency of information transmission to the control center. Furthermore, to obtain a holistic assessment of performance, self-reports are also included.

#### Visual Attention During Orientation at the MCI Scene

Visual attention has been suggested as a potential factor contributing to medical performance [17]. Consistent with the eye-mind hypothesis, gaze fixations are usually the focus of thought [23], which is why the interest in gaze behavior is increasing in research on medical decision-making and medical training [24,25]. Previous research demonstrated that perception and recognition skills improve with increasing medical experience [24]. Furthermore, visual attention differs with expertise in several medical tasks, such as surgical simulations and diagnostic decision-making [24,26]. Among other aspects, experts fixate on task-relevant cues more often and fixate less on task-irrelevant areas than novices [27].

Only a few studies have tested visual attention in prehospital settings thus far. Eye tracking while viewing photos of accident scenes revealed that MFRs fixated significantly longer on task-relevant cues, such as injured people or safety hazards, than non-MFRs [28]. However, the association between visual attention and expertise was less clear when tracking the eye movements of physicians and nurses watching a 2D video of an MCI [29]. Regardless of the number of years working in emergency medical services, all participants spent more time observing the patients who were severely injured. In addition, participants navigated through the scenes with less predetermined or structured patterns than initially expected. Another study assessed the visual attention of college students viewing a computer screen with photos of injuries and information boxes about airway, breathing, and circulation [30]. One group was trained in the *simple triage and rapid treatment* (START) triage algorithm, while the active control group was instructed in patient transport. After training, the START triage group fixated on relevant information boxes significantly faster. However, all of these studies relied on 2D stimuli presented on computer screens, and none tested visual attention of MFRs in 3D iVR MCI scenarios, although differences between 2D input and iVR have been documented for many cognitive and behavioral processes [7].

#### Accuracy and Speed of the Triage Process

Typical objective performance assessments in MCI training address accuracy and speed of the triage process [5,10]. Effective allocation of available resources and a well-decided sequence of patient care and transport are essential for managing MCI situations [4] and require the ability to triage patients correctly even under high stress. Triage accuracy refers to the correct choice of triage levels based on a specific triage algorithm. The START triage algorithm is an internationally known algorithm developed in the United States and used in several emergency medical services organizations [31]. According to START, patients are classified based on their injuries as green (minor priority), yellow (delayed priority), red (immediate priority), and black (dead or fatally injured). In addition to accuracy, triage speed is also considered crucial in the management of MCI situations so that patients with immediate priority receive medical treatment as quickly as possible [32]. In particular, triage accuracy, but also triage speed, have been used as effectiveness indicators for evaluating MCI training in previous studies [5]. Regarding iVR studies, only a few have included objective performance assessments within the virtual environment. These studies suggest that triage accuracy in iVR improves with more iVR training [33]. Furthermore, when comparing iVR to real-life exercises, MFRs had similar triage accuracy scores [4,34], but completed the triage process faster in iVR [4]. However, emergency physicians with more experience did not have higher triage accuracy scores in iVR than those with less experience [35]. This may be because the study did not aim to compare experts and novices, and those with less experience were already practicing residents. Therefore, the difference in practical experience was probably not large enough to be considered a typical expert-novice comparison.

#### Transmission of Information to the Control Center

One of the main problems commonly found during major incidents is poor communication with the control center [10,36]. For instance, communication problems, such as excessive radio traffic, can lead to several negative consequences, including increased mortality rates and reduced safety for MFRs at the scene [37]. Although information transmission to the control center is crucial to ensure proper coordination of all rescue services, studies measuring the quality [10] and efficiency (ie, accurate information in few words) of such radio messages are still missing. In Germany, the widely used *scene, safety, situation, and support* (SSSS) scheme serves as a standard for the assessment of onsite emergency situations [38]. This scheme provides a structured approach to assessing critical aspects of an incident. Sometimes referred to as the 3S scheme, with s*upport* not specifically named, the scheme assists MFRs in identifying and communicating all relevant information, especially potential hazards and environmental risks [39]. *Scene* refers to the assessment of the emergency site. In terms of safety, MFRs assess the risk to themselves and others. *Situation* refers to an estimation of the number of patients and evaluation of injury mechanisms. *Support* refers to the possible need for reinforcements and other emergency services, such as police and fire brigades [38]. On the basis of interviews and workshops with European MFRs, communication and the correct use of the scheme were identified to be key performance indicators in training for high-stress situations like MCIs [39]. While no studies seem to have evaluated the efficiency of information transmission, it was found that trained physicians reported more accurately than untrained physicians in a real-life MCI exercise [40].

#### Subjective Performance Ratings

A large part of MCI training studies evaluates the training effectiveness with self-rated indicators, such as self-ratings in knowledge and skills. For example, emergency medical technicians with more work experience rate their triage skills more favorably than those with less work experience [41]. However, general skill assessment does not necessarily relate to performance in a specific situation, and studies assessing self-rated MCI performance directly related to a previously completed simulation are still missing [5]. Not specific to MCI training but to medical education in general, previous research suggests that self-assessed performance has at least a low to moderate validity [42,43]. Self-rated indicators have the advantage of being easily implemented in all possible training modalities without any technical effort. Although objective performance assessment can be implemented in iVR, such assessment may be more complex in other training modalities, impairing potential comparison studies. If a global subjective performance indicator is accurate, it may be a low-threshold indicator for such studies. Including self-assessment alongside objective performance evaluation could also enhance the debriefing process by encouraging trainees to reflect on their performance and improve self-awareness [44]. However, comparisons between (global) subjective performance and objectively measured behavior in iVR MCI training are still lacking.

### Research Aims and Hypotheses

This study aimed to investigate whether different performance indicators that were used in previous MCI training studies can be effectively extended to MCI training in iVR. As a method of validation [17], indicators were tested with a focus on their ability to differentiate between different levels of expertise. For comprehensive testing, 2 indicators of expertise were used, including occupational group as a dichotomous indicator (MFR or non-MFR) and triage knowledge test scores as a continuous indicator. For a broad assessment, we investigated visual attention after arriving on scene, triage accuracy, triage speed, information transmission efficiency, and subjective performance. Overall, this study intends to provide insights into which indicators are suitable for incorporation into the design of effective iVR MCI training programs and MFR performance assessments. The following hypotheses were formulated: hypothesis 1a: with greater expertise, medical staff pays more attention to task-relevant information during MCIs; hypothesis 1b: with greater expertise, medical staff show greater triage accuracy during MCIs; hypothesis 1c: with greater expertise, medical staff complete the triage process faster during MCIs; hypothesis 1d: with greater expertise, medical staff transmit information more efficiently; hypothesis 2: attentional indicators demonstrate incremental value in the discrimination of different levels of expertise beyond performance indicators, such as speed, accuracy, and information transmission during triage; and hypothesis 3: subjective evaluation of one’s own performance is better with greater attention to task-relevant information, greater triage accuracy, faster triage time, and more efficient transmission of information.

The wording of 2 hypotheses has changed from the preregistration. Previously, they were “Hypothesis 1d: with greater expertise, medical staff transmits information more quickly and completely” and “Hypothesis 3: subjective evaluation of own performance is better with greater attention to task-relevant information, greater triage accuracy, faster triage time, and faster transmission of information.”

## Methods

### Study Design

In this quasi-experimental, multimethod study, participants (MFRs and non-MFRs) completed virtual MCI scenarios while their performance was assessed.

### Procedure

#### Overview

Interested people visited a website following the link or QR code provided on the recruitment materials. On the website, they received information on the study aims and procedure, provided informed consent, and answered a web-based preliminary questionnaire covering demographics, personal characteristics, and a triage knowledge test. The web-based questionnaire was answered before participants came to their appointment to shorten the time in the laboratory. At the end of the questionnaire, participants scheduled a 2-hour appointment in the VR laboratory. In the laboratory, participants filled out additional questionnaires, were shown the START triage algorithm, and engaged in an iVR familiarization scenario with people who were uninjured and at no accident site. During the familiarization, participants practiced information gathering and navigation within the virtual environment. The familiarization ended when participants announced that they feel sufficiently prepared. The main phase of the study involved 5 virtual MCI scenarios to cover the different performance levels of our sample, thus avoiding floor and ceiling effects and increasing the reliability of the measurements. The scenario order was randomized for each participant using a random number generator.

#### MCI Scenarios in iVR

The scenarios were built with the XVR software (XVR Simulation BV) and consisted of traffic accidents that varied in scenario complexity (Table 1; Multimedia Appendix 1 gives a detailed description). Tasks included gaining an overview of the situation, performing triage, and transferring all relevant information to the control center via radio messages.

**Table 1:** Scenario descriptions. After the familiarization scenario, the scenario order was randomized for each participant.

Scenario	Scene description	Number and triage levels of patients	Duration in minutes, mean (SD)
Familiarization	Quiet residential area, no accident, clear vision, middle of the day; 6 healthy people present, 3 of them sitting in cars	0 patients but healthy people to practice the iVR^a^ handling	5.78 (1.71)
1 (very low difficulty)	Accident with 2 cars on a countryside road, clear vision, middle of the day	3 green patients and 1 yellow patient	3.32 (1.03)
2 (low difficulty)	Accident with a car, a van, and a motorcyclist on a motorway, clear vision, cloudy day	1 green patient, 3 yellow patients, and 1 red patient	5.33 (1.37)
3 (medium difficulty)	Accident with a car and an SUV^b^ in a busy inner-city area with a crowd of bystanders around, clear vision, cloudy day	1 green patient, 3 yellow patients, and 2 red patients	5.06 (1.59)
4 (greater difficulty)	Expressway accident with 3 cars and a van; several bystanders, twilight, end of the day	1 green patient, 3 yellow patients, 3 red patients, and 1 black patient	7.28 (1.97)
5 (highest difficulty)	Expressway accident involving a bus and a truck, nighttime and fog	4 green patients, 6 yellow patients, 4 red patients, and 4 black patients	10.66 (3.08)

^a^iVR: immersive virtual reality.

^b^SUV: sports utility vehicle.

Participants were asked to remain in the starting position for the first 30 seconds during each scenario and were allowed to turn and look around. This time frame was used to assess initial attention processes measured via eye tracking. During the scenario, participants could use visual cues like wounds to select triage levels. Further information about the patients could be acquired verbally and was provided with the help of standardized audio tracks. For example, if participants inquired about the respiration rate, they would hear a prerecorded answer. Obtainable information included whether the patient could walk, whether the airways were clear, respiration rate, recapillarization time, presence of a radial pulse, existence of heavy bleeding, the patient’s responsiveness to simple instructions, and localization of injuries (eg, head injury and leg injury). The participants could announce their intention to perform actions, including clearing the airway, stopping heavy bleeding, and conducting triage. After each scenario, they filled out questionnaires and had the option to reread the START algorithm.

#### Hardware Description

**Figure 1:**
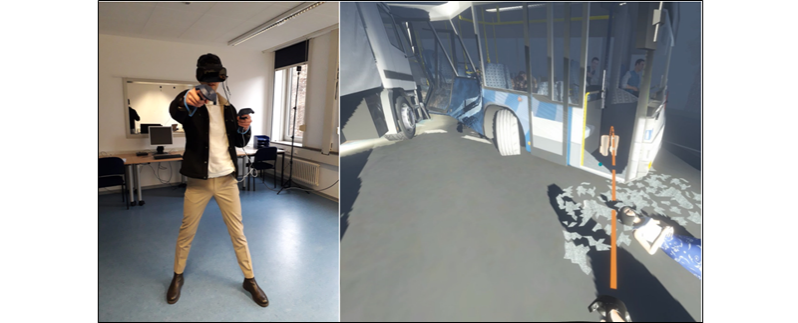
Immersive virtual reality (iVR) lab (left) and use of teleportation in iVR (right).

The Varjo Aero HMD had a display resolution of 2880×2720 pixels per eye at 90 Hz. Its field of view was 115° horizontally and 134° diagonally (at 12 mm eye relief), and the gaze data output frequency was 200 Hz. The HMD was connected to a laptop (ROG Strix G with Windows 10, Intel Core i7-9750H central processing unit at 2.60 Hz 2.59 GHz, NVIDIA GeForce TTX 2070 graphical processing unit, 32 GB of RAM) through the standard HMD cable and Varjo laptop adapter. Participants used the Varjo Aero HMD from Varjo Technologies Oy and could freely move within a 3×4-m size area. For greater distances, participants used controllers to teleport (Figure 1).

### Participants

Emergency services personnel, hospital personnel (physicians and nurses), and medical students (all semesters) were recruited to participate in this study. Inclusion criteria were a minimum age of 18 years, proficiency in German, and the absence of a hearing aid. Participants were recruited through social media, email distribution lists, flyers, and short presentations at local hospitals, emergency medical services, and university courses. Data collection lasted from February to October 2023. Of the 150 participants who filled out the web-based, preliminary questionnaire, 76 (50.7%) scheduled an appointment with our iVR laboratory and participated in the study. Participants were aged between 18 and 49 (mean 25.54, SD 6.01) years, and 59% (n=45) of participants were male. Among the 76 participants, 51% (n=39) were categorized as MFRs (n=36, 92% paramedics and n=3, 8% emergency physicians). The remaining 49% (37/76) non-MFR participants were mainly medical students (n=30, 81%), other medical staff not working in prehospital emergency settings (n=4, 11%), or both at the same time (n=3, 8%).

### Measures

#### Study Data

The data presented in this paper were derived from a larger project, which focused on investigating the stress dynamics and performance of MFRs in virtual MCI training. For detailed information of all measures and instruments used during the study, refer to the construct overview on the web [45].

#### Web-Based Preliminary Questionnaire

Demographic information included age, gender, profession, years of job experience in the medical sector, and prior MCI training in hours. In the case of profession, participants could choose multiple options from the following: emergency physicians, other physicians, paramedics, emergency medical technicians, medical students, or specify another option through an open text field. Prior MCI training was assessed with the following answering options: none, 1 to 5 hours, 5 to 10 hours, 10 to 20 hours, 20 to 30 hours, 30 to 40 hours, 40 to 50 hours, or >50 hours.

In terms of expertise, participants were classified as MFRs if they were emergency physicians or paramedics; otherwise, they were classified as non-MFRs. The second expertise measure was a triage knowledge test based on Cuttance et al [46] and adapted to the START algorithm. Participants had 10 minutes to assign triage levels to 20 case descriptions, after which the test ended, and the next page opened. One point was awarded for each correctly assigned color, resulting in a score range of 0 to 20.

Triage algorithm or algorithms. Participants were asked which triage algorithm they typically used in the field or in training sessions, including the option to select “none.”

Prior experience with iVR was assessed with the item “How much prior experience do you have with VR?” and a dropdown menu with 9 answering options from “no experience” to “daily use of VR.”

#### Visual Attention During Orientation in Each iVR MCI Scenario (Onsite)

Attention to task-relevant information was assessed using eye tracking in the first 30 seconds of each scenario. Eye-tracking data were collected using Varjo Base and analyzed with the software iMotions [47]. The gaze behavior was analyzed in terms of average fixation durations and number of fixations of the areas of interest (AOIs; [48,49]). AOIs included patients, vehicle impact zones, safety aspects, and a distractor (Multimedia Appendix 1 gives scenario descriptions). Within the iMotions software, AOIs were defined graphically, and the software automatically computed the fixation parameter [50].

#### Objective Triage Accuracy and Speed in Each iVR MCI Scenario (Onsite)

Triage accuracy was measured by the number of correctly assigned triage colors per scenario. The accuracy score for each scenario was divided by the number of patients in that scenario. The accuracy scores of all 5 scenarios were then averaged, leading to possible values between 0 and 1 (standardized Cronbach α=0.76).

Speed of triage was measured as the time from the start of the scenario to the completion of triaging the last patient (standardized Cronbach α=0.82). Note, however, that due to the different lengths of the scenarios, averaging with nonstandardized times would lead to a stronger weighting of the more complex scenarios with longer duration and a larger temporal variance. To avoid this, a standardization was performed: values were divided by the average triage speed of the specific scenario before being averaged. Consequently, values <1 signified a triage process faster than the average, while values >1 denoted a slower-than-average triage pace.

#### Information Transmission to Control Center in Each iVR MCI Scenario (Onsite)

To assess how quickly and completely medical staff transmit information, an efficiency measure was formed as a combination of completeness and speed. Participants’ radio messages during the scenarios were recorded and transcribed according to the content-semantic transcription by Dresing and Pehl [51]. A coding system based on the qualitative content analysis by Mayring [52] was then applied, with categories derived from the SSSS scheme [38] and added to a coding template. Next, 2 independent raters (university students specifically trained for this) analyzed the transcripts with the software MAXQDA (VERBI GmbH), without access to any information on the participants (interrater agreement: 97% for categorization and 93% for assessment of correctness). Discrepancies were resolved through discussion and, if necessary, together with a third rater (ASB). Statements were categorized, with a maximum of 1 point awarded for each of the 4 categories. Half a point was awarded for the category *scene* if participants only mentioned that it was a traffic accident, and a full point if more information was given (eg, the type of street or number of vehicles involved). Furthermore, a penalty for mistakes was implemented: 0.3 points were deducted for each error in the respective category. Regarding the category s*ituation*, an error was given if the number of patients was not stated correctly. In the 2 most complex scenarios, no penalty was applied if an estimate close to the correct number was reported (+2 or –2; eg, for scenario 4 with 8 patients, “almost 10” or “between 7 and 9”; n=4 cases). No penalty was given for incorrectly stated triage colors, as these are already covered by the triage accuracy variable. The scores of the 4 categories were added up (range: 0-4 points) and standardized based on the number of words in the radio message (ie, divided by the word count). This standardization accounted for unclear, redundant, and superfluous communication (possibly even in the same time by simply speaking faster), which poses a risk to the management of MCIs [53]. Finally, the average score across all 5 scenarios was calculated (standardized Cronbach α=0.83; range 0.03-0.15).

#### Subjective Performance of Each iVR MCI Scenario (Onsite)

Subjective performance was assessed with 2 items after each scenario: “How do you rate your performance in the last scenario?” (1=very bad and 10=very good) and “What school grade does your performance correspond to?” (scale: 1-6, with 1 being the best grade). We used 2 items to increase the reliability in capturing the construct. Before the items were averaged, the second item was inverted and transformed to match the 1 to 10 scale of the first item (Cronbach α=0.86):



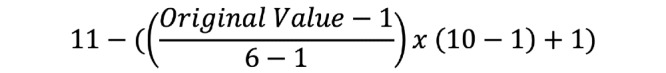



**(1)**

### Analyses

Analyses were conducted with R (version 4.3.2; R Core Team). Hypotheses 1a to 1d were tested with both expertise measures separately, distinguishing between MFRs and non-MFRs as well as using the triage knowledge test. For hypothesis 1a specifically, a multivariate ANOVA was used because of the multiple AOI categories. According to the Mardia test, the assumption of multivariate normal distribution was violated, but multivariate ANOVAs are robust to this specific violation, particularly in cases of homogeneous covariance matrices, which was given [54]. For hypotheses 1b, 1c, and 1d, independent *t* tests were performed to test for differences between MFRs and non-MFRs. For hypothesis 1b, the Welch *t* test was used because the Levene test indicated a violation of the assumption of homogeneity of variance. Hypotheses 1c and 1d were tested with the student *t* test. According to the Shapiro-Wilk test, the assumption of normality was violated testing hypotheses 1b and 1d. However, *t* tests are largely robust against this violation, especially because both group sample sizes were relatively similar and >30 [55,56]. The Bonferroni-Holm method was used to control for multiple testing in the *t* tests (hypotheses 1b-1d). Spearman rank-order correlations were used to examine the relationship between the knowledge test score and the outcome variables in hypotheses 1a to 1d, as they are robust to distributional violations and outliers. In addition, when correlations were significant, regression analyses were conducted to test the relationship while controlling for age and gender. We selected these 2 control variables because they are potential influencing factors in iVR performance situations [57,58]. For the regression analyses, independent variables and covariates were centered (for gender: –1=female and 1=male). Hypothesis 3 was tested with Spearman correlation analyses.

Outliers were defined as values that were 3 SDs above or below the mean. These values were winsorized (ie, set to mean +3 or –3 SDs, respectively) to reduce the influence of outliers on the results [59]. Analyses were conducted with adjusted outliers and with original variables to examine the robustness of results. When technical problems occurred during the assessment (eg, technical failure of the eye tracking or the microphone for radio message recording), the respective measure was excluded instead of excluding the entire dataset of the participant. Therefore, hypothesis 1a, which addressed eye-tracking data, was tested with a sample of n=71 for the AOIs *patients*, *safety*, and *vehicle impact zone*, and n=57 for the *distractor*. Hypothesis 1b and c, which examined triage behavior, were tested with n=74 and hypothesis 1d, which focused on information transmission, was tested with n=70 participants. There were no missing values for subjective performance (hypothesis 3).

### Ethical Considerations

This study was preregistered [60], approved by the ethics committee of the Faculty of Behavioral and Empirical Cultural Sciences at Heidelberg University (AZ Bae 2023 1/1), and carried out in accordance with the Helsinki Declaration on Ethical Principles of Research Involving Humans. To ensure transparency in the analysis process, the R Markdown file, containing the R code and all results, is available on the web [45]. Data was collected in a pseudonymized manner and subsequently anonymized for analysis. As an incentive, the participants received €25 (US $27.48 on Feb 1, 2023).

## Results

### Analyses

To capture 2 different measures of expertise, analyses were conducted with occupational group as a dichotomous measure (MFRs vs non-MFRs) and the triage knowledge test scores as a continuous measure. Means and SDs of performance indicators per scenario can be found in Table S1 in Multimedia Appendix 1.

### Demographics

The classification of expertise based on the profession was supported by the results of the knowledge test. MFRs had a significantly higher knowledge score than non-MFRs, *t_Welch_*_66.17_=–3.43, *P*=.001, *d*=0.79. More information on the groups can be found in Table 2. Most participants were not familiar with any triage system before this study (43/76, 57%; 34/43, 79% of those being non-MFRs). Some (27/76, 36%) participants knew the mSTART (modified START) algorithm, 12% (9/76) knew PRIOR (Primäres Ranking zur Initialen Orientierung im Rettungsdienst), and 1% (1/76) the START algorithm. Of the non-MFR group, 65% (24/37) had no prior MCI training, and 30% (11/37) non-MFRs had <5 hours. 3% (1/37) had up to 20 hours, and 3% (1/37) had up to 30 hours of MCI training. Regarding the MFRs’ prior MCI training experience, 8% (3/39) reported none, 31% (12/39) had up to 5 hours, 18% (7/39) had up to 10 hours, 18% (7/39) had up to 20 hours, 5% (2/39) had up to 30 hours, 8% (3/39) had up to 40 hours, 5% (2/39) had up to 50 hours, and 8% (3/39) had >50 hours.

**Table 2:** Description of expertise groups: medical first responders (MFRs) and medical students and other medical staff (non-MFRs).

Characteristics	MFRs (n=39)	Non-MFRs (n=37)
Age (y), mean (SD)	27 (7.15)	24 (4.07)
Gender: female, n (%)	11 (28)	20 (54)
Job experience (y), mean (SD)	6.53 (7.01)	1.62 (1.94)
Experience with iVR^a^, none or only 1-2 times, n (%)	33 (85)	34 (92)
Knowledge test score, mean (SD)^b^	13.46 (2.73)	10.89 (3.70)

^a^iVR: immersive virtual reality.

^b^The knowledge test score could range from 0 to 20.

### Visual Attention During Orientation and Expertise (Hypothesis 1a)

MFRs and non-MFRs did not differ significantly in their mean durations of average fixation in the 4 AOI categories of patients, vehicle impact zones, safety aspects and distractor, Pillai trace=0.03, *F*_4,52_=0.40, *P*=.81. Furthermore, there were no significant correlations between the knowledge test score and the average fixation duration in any of the 4 AOI categories (Table 3).

Regarding the fixation count, MFRs and non-MFRs did not differ across the 4 AOI categories (Pillai trace=0.15; *F*_4, 52_=2.37; *P*=.06). Again, the triage knowledge test was not significantly associated with the fixation count in any of the AOI categories (Table 4).

**Table 3:** Duration of average fixation (DOAF) of specific area of interest (AOI) categories across the 5 scenarios. Time in milliseconds: fixation duration was first averaged per scenario and then averaged across the 5 scenarios.

DOAF AOIs			Patients		Safety		VI^a^ zone		Distractor
Examples of AOI cues			All patients and no bystanders		Scenario 1: spilled oil; scenario 2: ongoing traffic; and scenario 5: ongoing traffic, spilled oil, broken glass		VI zones in all scenarios; in scenario 3 and scenario 4 mean value of 2 VI zones		Only in scenario 3: filming bystander
**Value per group, mean (SD)**									
	MFRs^b^	397.41 (105.55)		319.09 (105.69)		314.50 (80.39)		215.13 (92.28)	
	Non-MFRs	403.18 (106.97)		319.37 (91.20)		315.21 (92.61)		241.35 (118.76)	
**Correlation with triage knowledge test**									
	ρ	.06		–.10		.17		.23	
	*P* value	.61		.40		.15		.08	

^a^VI: vehicle impact.

^b^MFR: medical first responder.

**Table 4:** Number of fixations of specific area of interest (AOI) categories across 5 scenarios. Fixation count was summed up per scenario and then averaged across the 5 scenarios.

FC^a^ AOIs			Patients		Safety		VI^b^ zone		Distractor
Examples of AOI cues			All patients and no bystanders		Scenario 1: spilled oil; scenario 2: ongoing traffic; and scenario 5: ongoing traffic, spilled oil, and broken glass		VI zones in all scenarios; in scenario 3 and scenario 4 mean value of 2 VI zones		Only in scenario 3: filming bystander
**Value per group, mean (SD)**									
	MFRs^c^	12.51 (4.43)		8.90 (5.46)		8.84 (3.29)		2.73 (2.13)	
	Non-MFRs	12.76 (4.30)		11.51 (6.47)		7.71 (2.80)		3.85 (2.36)	
**Correlation with triage knowledge**									
	ρ	–.09		–.16		.02		.04	
	*P* value	.46		.18		.89		.76	

^a^FC: fixation count.

^b^VI vehicle impact.

^c^MFR: medical first responder.

### Triage Accuracy and Expertise (Hypothesis 1b)

**Figure 2:**
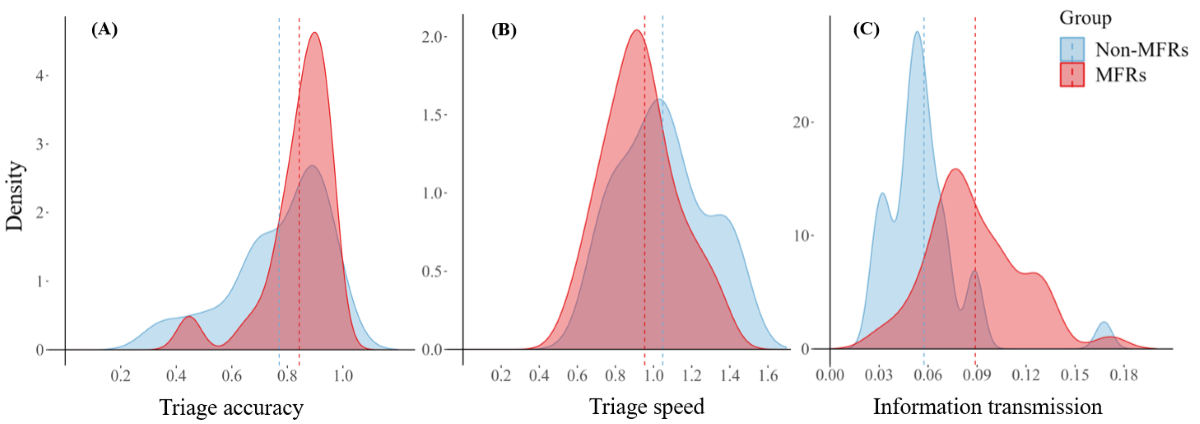
(A) density plots per group for triage accuracy, (B) triage speed, and (C) information transmission efficiency. The dashed lines mark the average per group. MFR: medical first responder.

MFRs had a significantly higher triage accuracy than non-MFRs (*t_Welch_*_61.62_=–2.04; *P*=.02 [Bonferroni-Holm corrected *P*=.045]; *d*=0.48). On average, MFRs triaged 84% (SD 12%) of the patients correctly while non-MFRs triaged 77% (SD 18%) correctly (Figure 2A depicts the distributions).

Furthermore, a higher knowledge test score was associated with significantly higher triage accuracy (Spearman ρ=0.40; *P*<.001). A multiple regression analysis was used to test this relationship while controlling for age and gender. More prior triage knowledge significantly predicted higher triage accuracy during the scenarios (*b*=0.02; SE 0.005; *P*=.002), while age (*b*=–0.001; *P*=.67) and gender (*b*=0.005; *P*=.89) were not significant predictors. The model explained 9% of the variance (adjusted *R*^2^) (*R*^2^=.13; *F*_3,70_=3.45; *P*=.02). However, according to the Shapiro-Wilk test, the assumption of normal distribution of the residuals (*P*<.001) was violated. Therefore, bootstrapping with 5000 draws was used to test the stability of the results. Again, prior triage knowledge significantly predicted triage accuracy during the scenarios (*b*=0.02, SE 0.00, 95% CI 0.01-0.03).

### Triage Speed and Expertise (Hypothesis 1c)

MFRs completed the triage process significantly faster than non-MFRs (*t*_72_=1.79; *P*=.04 [Bonferroni-Holm corrected *P*=.045]; *d*=0.42). On average, MFRs required 95% of the average triage speed, whereas non-MFRs needed 5% more than the average (MFRs: mean 0.95, SD 0.23; non-MFRs: mean 1.05, SD 0.23; Figure 2B depicts the distributions). The knowledge test score was not significantly associated with triage speed (Spearman ρ=–.04; *P=*.72).

### Information Transmission and Expertise (Hypothesis 1d)

MFRs transmitted information significantly more efficiently than non-MFRs (MFRs: mean 0.09, SD 0.03, and non-MFRs: mean 0.06, SD 0.03; *t*_68_=–4.74; *P*<.001 [Bonferroni-Holm corrected *P* <0.001]; *d*=1.13) (Figure 2C depicts the distributions). However, there was no significant correlation between the knowledge test score and efficiency of information transmission (Spearman ρ=0.19; *P=*.11).

Explorative analyses were conducted with information transmission scores and word counts separately. Averaged across the scenarios, MFRs did not transmit significantly more correct information (MFRs: mean 2.15, SD 0.80, and non-MFRs: mean 2.07, SD 0.72; maximum score=4; *t*_68_=–0.44; *P*=.33; *d*=0.11). However, MFRs transmitted information in fewer words (MFRs: mean 32.34, SD 20.99, and non-MFRs: mean 46.09, SD 23.68; *t*_68_=2.57; *P*=.006; *d*=0.62). Knowledge test scores were not significantly correlated with the number of correct information transmissions (Spearman ρ=–0.05; *P*=.67) or information transmission length (Spearman ρ=–0.13; *P*=.29).

### Testing the Incremental Value of Attention (Hypothesis 2)

As reported for hypothesis 1a, the attentional indicators assessed with eye tracking did not discriminate between different levels of expertise. Accordingly, testing the incremental value of visual attention indicators beyond the other performance indicators for distinguishing levels of expertise (hypothesis 2) was inapplicable.

### Subjective Performance and Objective Performance Indicators (Hypothesis 3)

**Figure 3:**
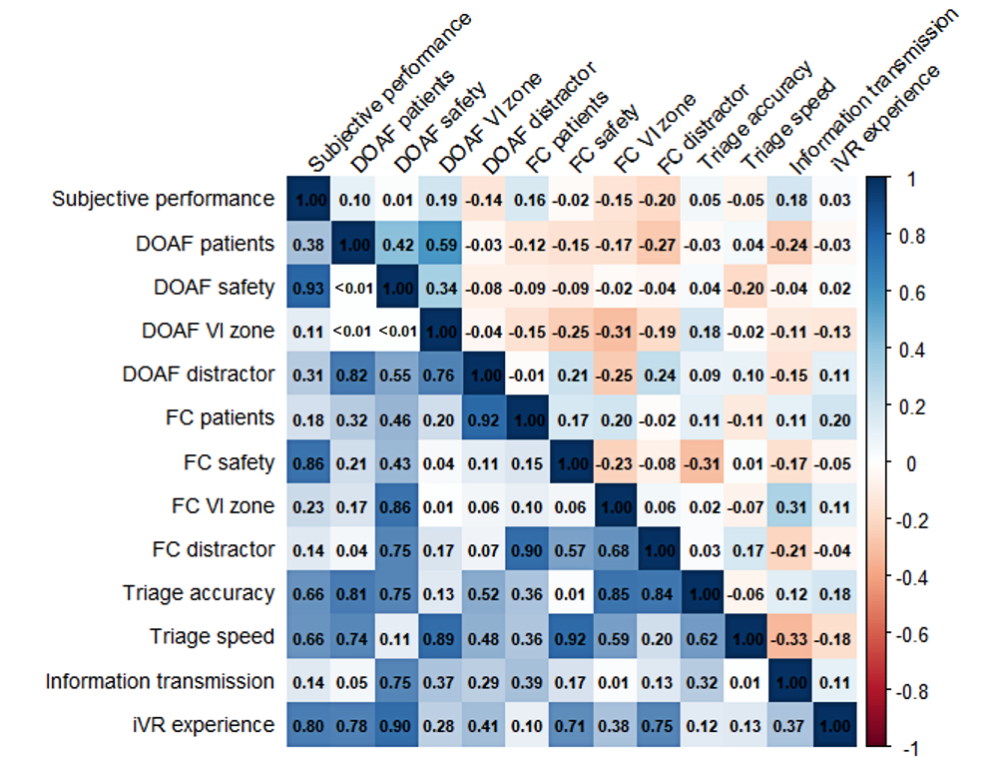
Spearman correlation analysis of the performance indicators and immersive virtual reality (iVR) experience (above diagonal: ρ values, below diagonal: *P* values). iVR experience refers to prior iVR experience as assessed in the preliminary questionnaire; prior iVR experience was not winsorized because of the ordinal data structure. DOAF: duration of average fixation; FC: fixation count; VI: vehicle impact.

There were no significant correlations between subjective performance (mean 6.77, SD 1.13) and any of the objective performance indicators (all *P*>.05; Figure 3). Conversely, significant associations were observed between some objective performance indicators, including between greater triage speed and less efficient information transmission (ρ=–0.33; *P*=.01), and between less visual attention to safety cues (as measured by fixation count) and greater triage accuracy (ρ=–0.31; *P*=.01).

### Explorative Analyses

Exploratively, we tested whether prior iVR experience was associated with any of the examined performance indicators. For both MFRs and non-MFRs, the overall median value was 2 (ie, tried iVR 1-2 times). Prior iVR experience did not correlate with any performance indicator (all *P*>.05; Figure 3).

## Discussion

### Principal Findings

The prehospital triage process is a central aspect of managing MCIs, and if done correctly, leads to an efficient allocation of treatment and transport. As MCI real-life exercises are resource-intensive and therefore infrequently conducted, iVR has emerged as a new triage training method [5]. Due to the novelty of such training, systematic evaluations of potential indicators that can be used to assess performance are still needed. This study aimed to provide such an evaluation. The objective performance indicators of triage accuracy and speed, as well as information transmission efficiency, significantly differentiated between MFRs and non-MFRs. In addition, higher triage accuracy was significantly associated with more prior triage knowledge, even when controlling for age and gender. Interestingly, however, visual attention did not differ with the level of expertise. Furthermore, in contrast to the third hypothesis, subjective performance was not correlated with any other performance indicator. Next, we discuss possible explanations as well as future directions for deriving meaningful performance indicators in iVR MCI training.

### Visual Attention During Orientation

Visual attention represents a relatively novel potential performance indicator that has received limited attention from research in prehospital contexts. In other medical fields, studies using eye tracking have already identified differences in visual attention between experts and novices [17,24,26]. In this study, we found no significant associations between visual attention and expertise, which aligns with findings based on 2D videos of an MCI [29]. However, the results stand in contrast to 2 studies using 2D, prehospital images as stimuli [28,30]. Loth et al.[30] showed that after START triage training, participants directed their attention more swiftly to triage information boxes. The transferability of such findings to a 3D virtual scenario without information boxes remains uncertain [30]. In the second study, MFRs and novices differed significantly in their visual attention when looking at MCI photos without information boxes [28]. Several factors could explain the discrepancies. First, the former study used a purely 2D examination, which may not translate seamlessly into 3D. In contrast, iVR offers greater ecological validity for natural gaze behavior by providing a larger field of view and the ability to visually explore the environment by moving the head [49]. Moreover, the stereoscopic visualization in iVR enhances depth perception through binocular cues [61]. Previous research has demonstrated that 3D input can significantly alter attentional processes compared to 2D input [62], although it has not yet been directly compared in the context of MCI scenarios. The additional visual cues in the iVR scenarios of this study may have enabled non-MFRs to more quickly identify relevant AOIs, thereby reducing differences between MFRs and non-MFRs. Second, it is also possible that MFRs had to exert more effort in filtering out irrelevant visual stimuli than they would with 2D input, which could diminish potential differences between MFRs and non-MFRs. Furthermore, the former study [28] included true novices, whereas this study included non-MFRs with medical knowledge (primarily medical students), suggesting a possible power issue due to minimal effects between these groups, necessitating a larger sample size. Alternatively, it is plausible that both groups in this study possessed a minimum level of medical expertise necessary for effective visual attention, and effective visual attention remains relatively stable with additional triage knowledge.

Furthermore, for MFRs, visual attention, especially toward nonpatient information, such as safety threats and distractors, may be less critical than in professions such as law enforcement or military, where constant threat awareness is an integral part of training. For instance, police officers with specialized training have exhibited superior visual search behavior for threats and overall performance in real-life exercises compared to their counterparts without such training [63]. Another possibility is that differences in visual attention may only manifest when observing individual patients and their injuries, whereas our study primarily involved participants viewing a broad overview of an accident scene during eye-tracking measurements. Finally, it is worth considering that alternative visual attention indicators may offer more nuanced insights. The selection of appropriate eye-tracking measures is crucial, as certain fixation and saccade metrics or scan paths may be more applicable for specific purposes than others, depending on the task and study population [64]. This study used 2 eye-tracking measures often used in studies on visual attention in medical contexts [21,24], the number and length of fixations in predefined areas of interest (ie, patients, safety aspects, vehicle impact zone, and distractor AOIs). While fixation-derived metrics can be used as a measure for visual attention and processing, saccade-derived metrics could provide further insights into visual search strategies [24]. Furthermore, experts may exhibit distinct eye movement patterns and rapidly develop a holistic view of the scene, as observed in other medical domains [24].

### Triage Accuracy and Speed

During real-life MCI exercises, triage accuracy and speed have proved to be useful as performance indicators, although usually not comparing different levels of expertise but prepost improvements after training [65-67]. Other studies used the indicators to compare different training methods [4,68-70]. Triage accuracy and speed have also been used in iVR settings [4,33,35], with evidence suggesting that with more iVR training, participants become better and faster at triaging in virtual MCI situations [33].

The results of this study suggest that accuracy is a suitable performance indicator to differentiate between different levels of expertise, with a medium effect size in terms of profession (MFRs vs non-MFRs) and a medium to large effect size in terms of triage knowledge [71]. These results are consistent with a previous study that found paramedic students to be as good at triage accuracy in a real-world exercise as in an iVR scenario [4]. Conversely, a previous iVR study based on 360° video recordings found a less clear relationship between triage accuracy and expertise, as there were no significant differences in triage scores between residents and attendings or between those with and without prior MCI experience [35]. The medium rather than large effect size in the comparison between MFRs and non-MFRs may be because the non-MFR group were not true novices but also had some medical knowledge. In addition, participants were able to reread the START triage algorithms before starting the scenarios, possibly reducing the difference in triage accuracy performance.

In addition, this study suggests that speed can serve as an indicator to discriminate between MFRs and non-MFRs, as MFRs completed triage faster with a medium effect size [71]. However, caution should be exercised when comparing this marker to real-life exercises, as previous studies suggest that triage in virtual MCI scenarios may occur faster than in real-life scenarios [4]. This difference is likely due to differences in locomotion and information retrieval. In this study, participants were able to teleport with their controllers, allowing them to quickly cover large distances. Walking, as a method of locomotion, would be closer to real life but would require significantly larger training areas. Future studies could compare MFRs and non-MFRs in iVR systems with natural locomotion and more realistic patient information retrieval to test whether the differences between the groups are similar or greater than in this study.

### Information Transmission

The transmission of information to the control center is crucial for the effective coordination of rescue services and thus for the management of large-scale operations. As depicted in the SSSS scheme, the rapid and clear transmission of information regarding the MCI scene, safety hazards, patient information, and the need for additional support is essential for mobilizing necessary reinforcements [38]. To our knowledge, this study was the first to record radio messages and assess information transmission efficiency as a performance indicator differentiating between different levels of expertise in iVR MCI training. MFRs transmitted information more efficiently than non-MFRs (large effect size [71]), yet no correlation with the triage knowledge test score was observed. Furthermore, exploratory tests revealed that MFRs did not transmit significantly more accurate information but managed to do so using fewer words. These results suggest that a certain level of theoretical triage knowledge, that both groups seem to have had, is sufficient to facilitate MFR communication. However, the ability to quickly convey relevant information may improve with practical field experience.

The finding that MFRs transmit information more efficiently is consistent with a previous non-iVR study, which found that trained physicians were better at following a reporting scheme in a real-world simulation than their untrained counterparts [40]. However, the radio messages in the former study were rated by observers only in 3 categories: not performed, incomplete, and complete. In this study, the radio messages were analyzed in more detail. With the proposed method, trainees could receive more specific feedback in addition to their efficiency score, including which SSSS categories they missed or inaccurately reported. Furthermore, information transmission as a performance indicator holds promise for seamless integration into iVR, given the prevalent incorporation of microphones in iVR headsets and the increasing feasibility of automated evaluations through real-time transcription and artificial-intelligence applications. Future research may want to compare radio transmissions from iVR exercises with real-life exercises and actual MCI radio communications, providing further insight into differences in communication effectiveness and situational awareness, including the recognition and prioritization of hazards. In an Austrian study with 7 firefighters and 6 paramedics, participants underwent a 1-minute reporting phase following either an MCI scenario in iVR or nonimmersive VR on a computer screen [72]. In the former study, the information given during the reporting phase was analyzed regarding situational awareness, the anticipation of consequences, and the communication of actions. There was no significant difference between the 2 training modalities. This highlights the utility of verbal information reporting as a performance indicator not only within iVR contexts but also across various training modalities.

Nevertheless, future training should consider the locally used reporting schemes. In the United Kingdom, for instance, the METHANE (a mnemonic for “major incident declared; exact location; type of incident; hazards; access; number and type of casualties; emergency services present and required”) framework is commonly used as a guideline for reporting information [36]. While the SSSS scheme greatly overlaps with METHANE, certain aspects were not particularly explored in this study, such as the exact location and access possibilities. In this study, participants started at the accident site instead of arriving there using a virtual ambulance vehicle and also received no detailed information about the site beforehand. Such aspects must be considered in studies using the METHANE scheme.

### Subjective Performance

Self-reported performance did not correlate with the applied objective performance indicators, which contradicts previous research suggesting that self-assessed performance validity is at least low to moderate in medical education [42,43]. This discrepancy may stem from the fact that individuals often base their perception of abilities on comparisons [73]. In terms of self-evaluation, people commonly compare themselves to others (social comparisons) or to their own past performance (temporal comparisons) [73]. However, in this study, the participants did not witness other trainees’ performances, limiting social comparison options. In addition, comparing one’s own progress in performance may have been complicated by the scarcity of MCI training, particularly because the non-MFR group lacked prior MCI training experience. It is also possible that asking about a specific behavior, such as self-rated information transmission performance, might have resulted in an estimate closer to the specific objective indicators. While we assessed global self-rated performance, future studies could test whether specific self-rated performance assessments correlate with objective indicators.

Overall, the findings underscore the significance of integrating objective indicators, providing trainees with benchmarks to assess their performance. Still, self-evaluations remain important because they help trainees reflect on their performance and enhance their self-awareness [44]. Furthermore, comparing objective and subjective performance could potentially mitigate biases, such as the tendency for women to underestimate themselves compared to men [43].

### Limitations and Future Research

This study included a German sample of participants that were, on average, relatively young. Consequently, the generalizability of these findings to older populations and those from other countries should be tested in future research. For instance, previous iVR research found that cohorts over the age of 40 years had lower triage accuracy than younger cohorts [35]. This could potentially be attributed to greater familiarity with learning technologies.

This study was based on the START triage algorithm because it is well known internationally, and several triage algorithms are based on it [74]. However, in Germany, the learned triage algorithm varies depending on the rescue organization and region; thus, most of our sample was unfamiliar with START beforehand. Nevertheless, it is quite similar to the mSTART algorithm [74], which was the most frequently known algorithm in our sample. Future studies could program scenarios to initially allow selection of the learned algorithm, through which the scenario would then proceed, as in a previous study [75].

Furthermore, research on visual attention in prehospital settings is still in its infancy. Larger studies considering various attention and eye-tracking indicators, such as gaze movement patterns, would be beneficial in this regard. In this study, we investigated visual attention after arriving on scene and therefore only used the first 30 seconds. For standardization purposes, participants stayed at the starting position during this time. However, visual attention during the exploration of the scene or even during the whole scenario could also be an interesting indicator and more representative of behavior at real MCIs.

In this study, the ability to interact with patients was limited due to technical constraints of the software. In contrast to 360° videos, a major strength of this study was that participants were free to move around, which required them to decide how to navigate the environment, gain a full overview, and ensure that all patients were located. However, other than moving freely and using the controllers to open car doors, other actions, such as obtaining patient information, relied solely on visual and auditory cues as well as verbal information acquisition. Recent advances in iVR MCI software have introduced additional interaction features, including the use of a virtual toolkit with controllers, haptic feedback through vibrating controllers when taking a pulse, and patients responding to simple commands through automated speech recognition [15]. As the need for verbal requests to obtain patient information may have reduced immersion, future training may benefit from the use of iVR MCI tools with more interactive features than those available in this study.

In addition, the assessment of expertise should be examined in a more nuanced manner. We used the participants’ professions as well as a triage knowledge test as expertise indicators to cover the expertise construct in a broad manner, including theoretical knowledge and practical experience. In contrast, previous studies often only used one indicator, such as years of experience [29] or profession [28]. Still, measuring expertise is challenging and likely depends on numerous factors, such as years of experience (full or part-time), exposure to MCIs, and the amount of MCI training. Future studies could delve deeper into identifying measures suitable for assessing expertise and perhaps propose a composite measure encompassing multiple factors.

### Practical Implications

This study’s results highlight the suitability of iVR scenarios for MCI training and performance assessment. With its resource-efficient implementation, iVR training offers a valuable opportunity to supplement current MCI training. Although nonimmersive VR on a computer monitor may be an even more affordable option, immersive training is associated with learning gains, increased enjoyment, and improved concentration [76,77]. While initial costs for hardware, software, and personnel occur, iVR becomes less expensive with repeated use compared to real-life exercises, which require ongoing organizational and financial resources [4]. In addition, iVR software can reduce costs associated with observers by automating performance assessments, facilitating self-directed training, and allowing trainers to focus on higher-level observations. Because previous research has shown that MCI performance declines over time, MCI training should be conducted more frequently than once a year to ensure high-quality triage [67]. In this context, iVR training and performance assessment could provide a valuable supplement to enable regular MCI training for large numbers of first responders. The integration of iVR into standard training curricula could begin with theoretical instruction [67], followed by regular iVR training, which could also be used to prepare MFRs for large-scale, real-life exercises.

In particular, triage accuracy, speed, and information transmission seem to be effective performance indicators for possible incorporation into iVR MCI training, complementing each other by providing insights into various aspects of overall performance. These indicators could be seamlessly integrated into future iVR learning programs to automate performance evaluation, thereby reducing the workload on trainers and allowing them to focus on higher-order evaluations, such as general procedures. The tested indicators are all suitable for individual MCI training but may also be applied in team training and could even be enhanced with novel team performance indicators, such as position and movement tracking [78]. Furthermore, the integration of real-time performance measurement into a (smart) scenario control could be valuable. On the basis of live data, the scenario difficulty could be dynamically adjusted by either the trainer or artificial intelligence to meet the individual needs of trainees.

### Conclusions

Overall, iVR proved to be a valuable tool for assessing the performance of MFRs in MCIs scenarios. The performance indicators triage accuracy, triage speed, and information transmission can be extended to MCI training in iVR and capture multiple aspects of MCI performance. While visual attention did not function as a valid performance indicator in this study, future research might further explore visual attention as a potential indicator by examining other aspects, such as gaze patterns. Overall, iVR could be integrated into current MCI training curricula to provide objective and potentially automated performance assessments and allow for more frequent practice.

## Appendix

Multimedia Appendix 1 Descriptions of immersive virtual reality scenarios and performance per scenario.

## Abbreviations

AOI: area of interest
HMD: head-mounted display
iVR: immersive virtual reality
MCI: mass casualty incident
MFR: medical first responder
mSTART: modified simple triage and rapid treatment
PRIOR: Primäres Ranking zur Initialen Orientierung im Rettungsdienst; Primary Ranking for Initial Orientation in Emergency Medical Services
SSSS: scene, safety, situation, and support
START: simple triage and rapid treatment
