# Supplementary material for: Mass Casualty Incident Training in Immersive Virtual Reality: Quasi-Experimental Evaluation of Multimethod Performance Indicators
Source: J Med Internet Res. 2025 Jan 27;27:e63241. doi: 10.2196/63241 (PMC11811659; doi:10.2196/63241)
Supplement: Multimedia Appendix 1 [file jmir_v27i1e63241_app1.pdf]

Multimedia Appendix: Descriptions of immersive virtual reality scenarios and performance per scenario

Scenario Descriptions

Triage color categories: Green = minor injuries; yellow = patient's transport can be delayed; red = immediate intervention and transport needed; black = deceased (see the START triage algorithm for further information)

Scenario 1 (Very Low Difficulty)

- Accident on countryside road (clear vision, middle of the day)
- Two cars with four people involved in frontal crash (one car left its lane and crossed into incoming traffic)
- The cars have minor damage (clearly visible brake marks indicate the cars collided at low speed)
- No bystanders or other vehicles around
- Traffic was stopped by the police
- Spilled oil
- Triage
    - Four injured in total
    - Three individuals are green, one yellow

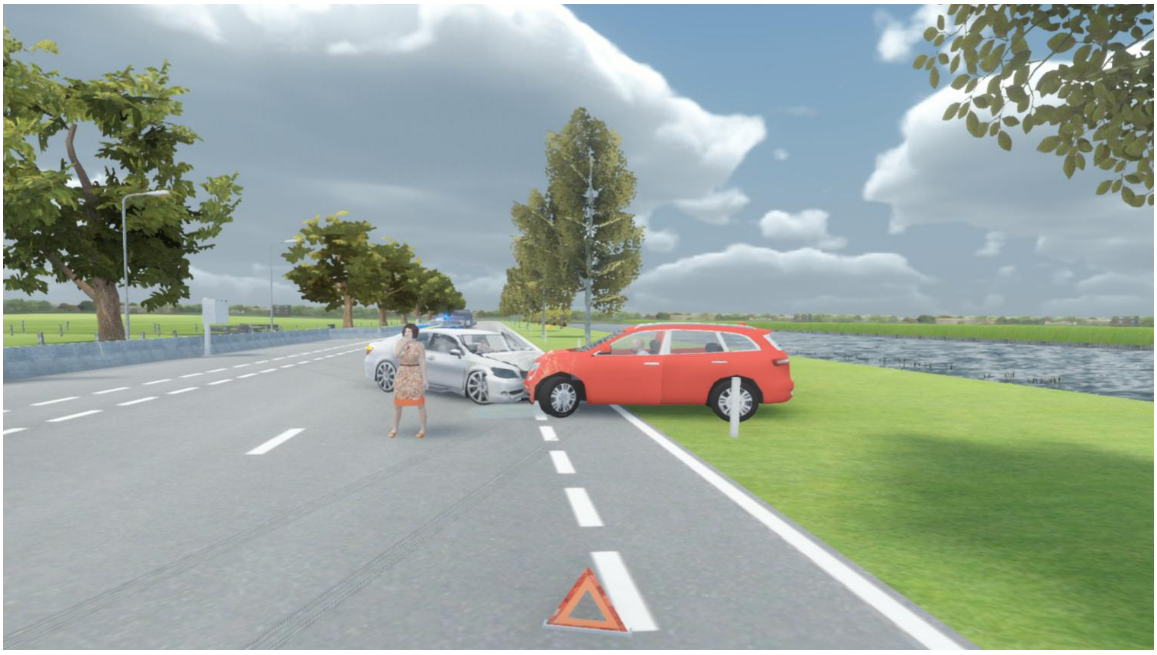

Scenario 2 (Low Difficulty)

- Accident on a motorway (clear vision, cloudy day)
- A car and a van were involved in a frontal crash (one car left its lane and crossed into oncoming traffic). Additionally, a motor cyclist crashed into one of the cars and fell far from the site
- The vehicles show larger degrees of damage; light smoke emanates from the crash zone
- The accident site has already been secured; traffic passes by slowly but quite close to the accident scene
- Spilled oil around the motorcycle
- One uninvolved car with three bystanders (a father with his son and a toddler daughter left in her car)
    - The father and the boy attend to two injured people while the toddler is continuously screaming (distractor)
- Triage
    - Five injured in total (two from each car + a motor cyclist)
    - One person from the cars is red (removed from the vehicle, attended to by the bystanders)
    - The other person from the car is yellow (also sitting outside the car)
    - In the van, one person is yellow and the other passenger green
    - The motor cyclist (far away from the site) is yellow

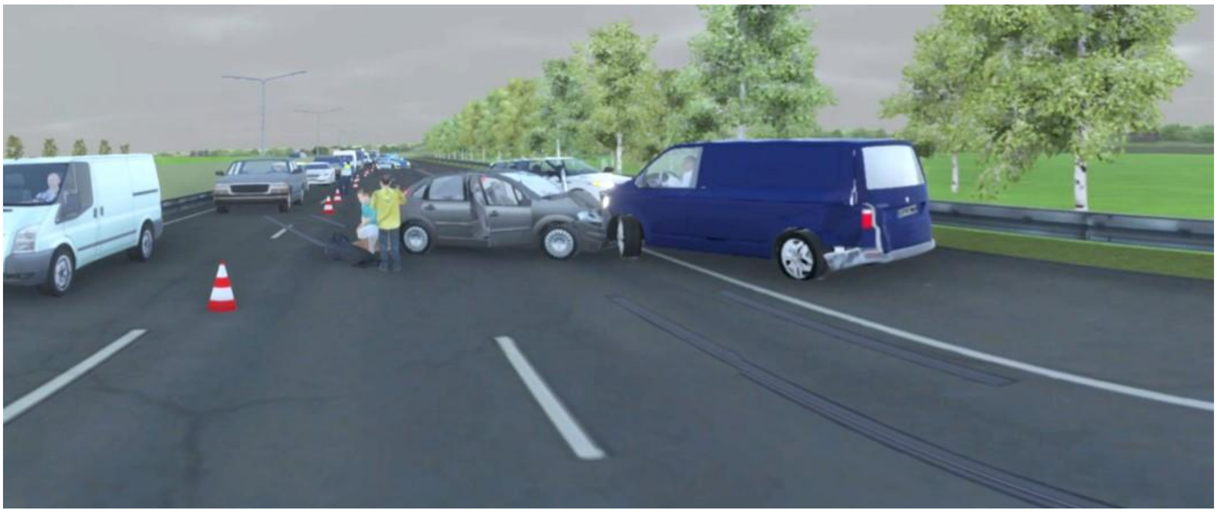

Scenario 3 (Medium Difficulty)

- One car has been rear-ended (with an angle) in a busy inner-city area. The hit car skipped onto the sidewalk and collided with pedestrians, one of which is stuck under the car
- The street is narrow and many bystanders stand around. High levels of noise come from the crowd. One bystander accuses the MFR of working too slowly; another bystander films the scene with her smartphone (distractors)
- The police stopped the traffic
- Triage
  - Six injured in total
  - The person under the car is red
  - The person who caused the accident was thrown from their SUV (no seatbelt worn), also red. The driver's daughter is screaming for help (green). Another passenger in the vehicle is yellow.
  - The driver of the other car is yellow
  - One pedestrian is yellow

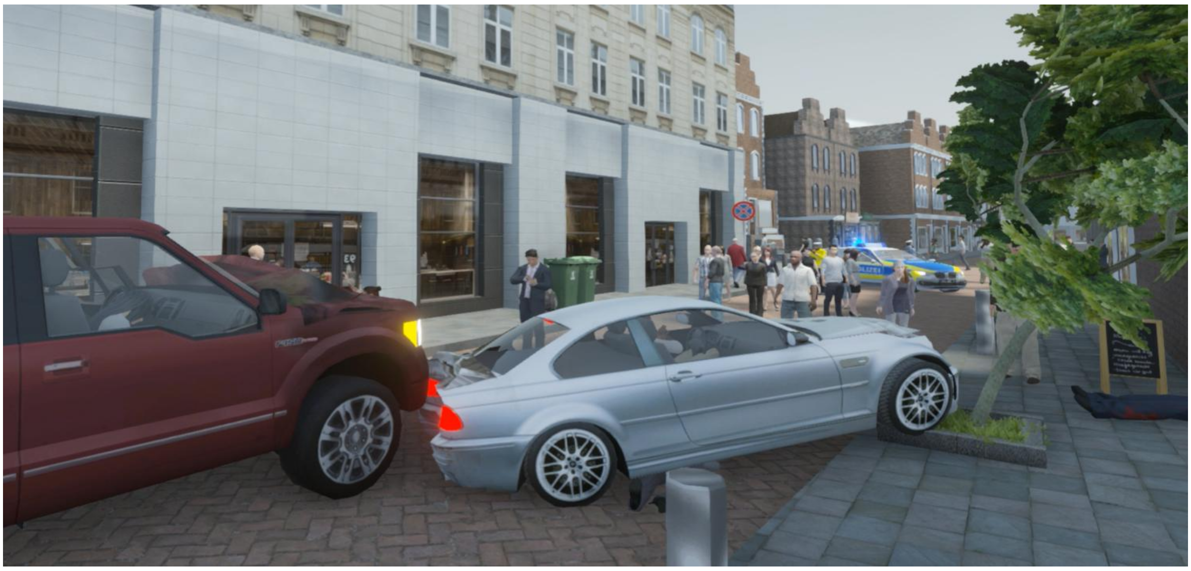

Scenario 4 (Greater Difficulty)

- Express way accident involving four vehicles in total
- Twilight
    - One van with only a driver
    - Three cars: one with three passengers (one adult male, one adult female who is pregnant, one child), one with two passengers (one adult, one child), another one with two passengers (two adults)
- (Almost) all individuals were removed from the cars
- Heavy damage to the van
- Many bystanders around; a high level of noise comes from the crowd and traffic; a bystander toddler was left in one of the bystander cars and cries loudly (distractors).
- A large dog is walking around unleashed and barking
- Oil spill, broken glass on the floor, van is smoking at the front (light smoke)
- Triage
    - Eight injured in total
    - Van driver black
    - Family: driver (male) yellow, pregnant woman yellow, child green
    - One of the other cars: adult driver red and child yellow
    - Other car: both adults red

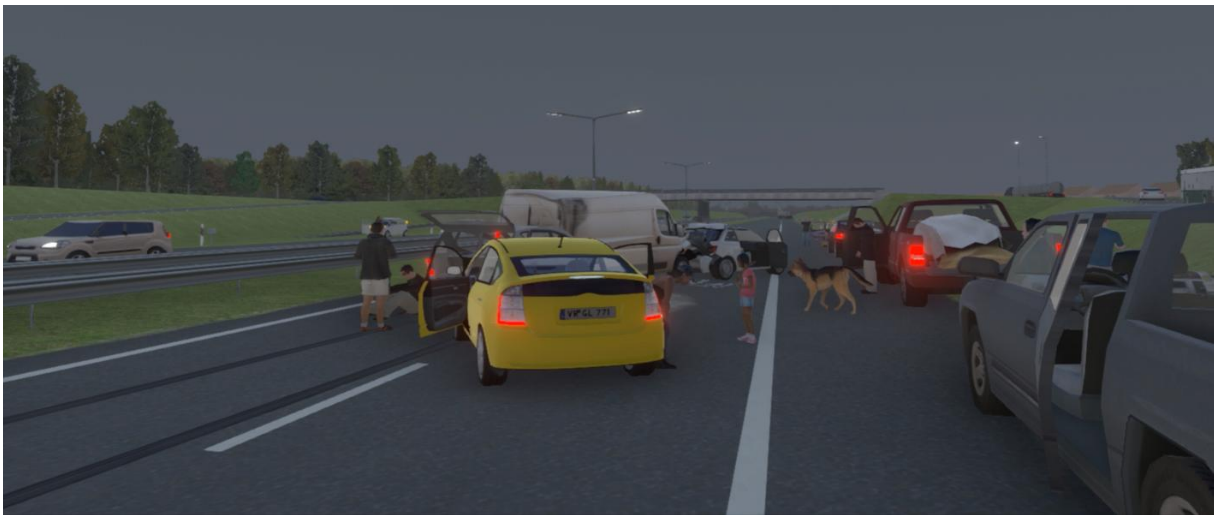

Scenario 5 (Highest Difficulty)

- Express way accident involving a bus and truck
- Night-time and fog
- Oil spilled and dark smoke emanates, with broken glass on the pavement
- The accident site is already secured; traffic passes by relatively close to the accident scene; the fire brigade has already set up floodlights
- Some people are screaming (distractor)
- Triage (18 people involved)
    - Truck driver black
    - Patients already outside of the bus: two black, two red, four green
    - Patients inside the bus: one black, six yellow, two red

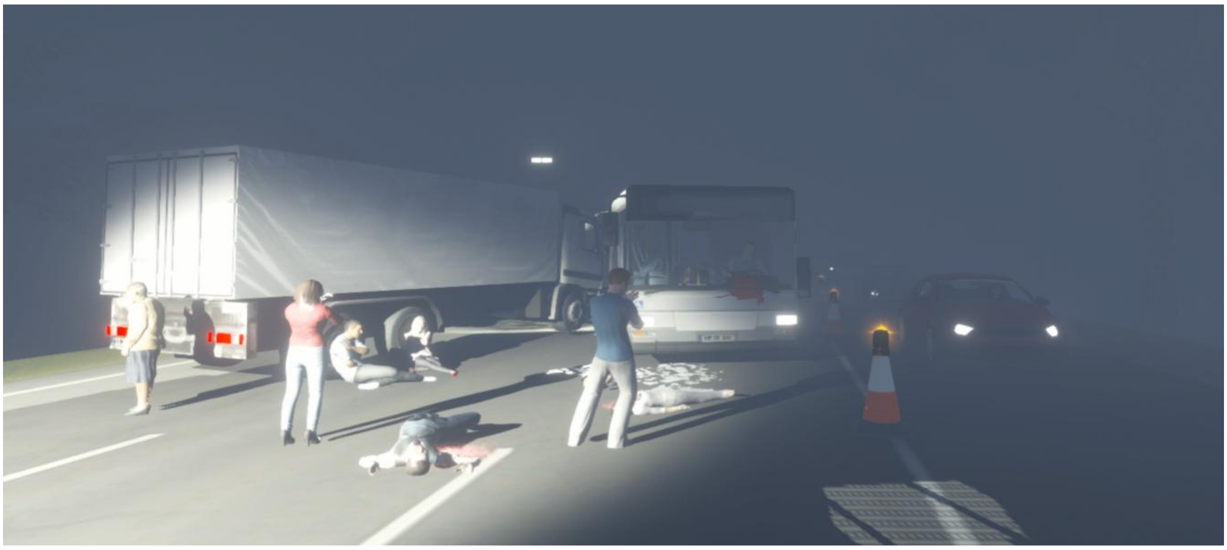

Performance per Scenario

Supplementary Table 1. Means and standard deviations of the performance indicators per scenario

Indicator	Scenario 1	Scenario 2	Scenario 3	Scenario 4	Scenario 5
DOAF patients	372.85 (138.62)	457.26 (237.30)	451.71 (193.69)	382.55 (214.41)	336.89 (113.96)
DOAF Safety	316.46 (200.61)	338.82 (138.54)	Not appl.	Not appl.	306.31 (95.20)
DOAF VI zone	271.77 (97.65)	302.97 (118.61)	280.13 (92.32)	374.25 (267.63)	341.27 (164.07)
DOAF distractor	Not appl.	Not appl.	233.33 (124.27)	Not appl.	Not appl.
FC patients	13.82 (7.81)	5.81 (4.43)	12.60 (5.96)	8.25 (5.58)	23.72 (11.98)
FC safety	1.66 (0.81)	12.71 (8.77)	Not appl.	Not appl.	10.83 (5.42)
FC VI zone	12.39 (9.86)	6.11 (4.21)	11.43 (5.67)	4.40 (3.13)	5.83 (3.47)
FC distractor	Not appl.	Not appl.	3.35 (2.34)	Not appl.	Not appl.
Triage accuracy st.	0.78 (0.28)	0.80 (0.26)	0.83 (0.19)	0.85 (0.19)	0.78 (0.18)
Triage accuracy non-st.	3.11 (1.11)	3.99 (1.30)	4.97 (1.15)	6.84 (1.52)	14.00 (3.25)
Triage speed st.	1.00 (0.37)	1.00 (0.26)	1.00 (0.27)	1.00 (0.32)	1.00 (0.33)
Triage speed non-st.	2.56 (0.94)	3.99 (1.03)	3.89 (1.04)	5.62 (1.78)	9.02 (2.96)
Info. transm. efficiency	0.11 (0.06)	0.07 (0.05)	0.07 (0.04)	0.07 (0.03)	0.05 (0.03)
Info. transm. word count	27.40 (21.80)	39.61 (30.70)	38.35 (29.52)	40.76 (29.87)	47.37 (30.10)
Info. transm. scores	2.16 (0.85)	2.05 (0.89)	2.10 (0.88)	2.18 (0.97)	2.02 (0.89)
Subj. performance	7.40 (1.40)	7.07 (1.52)	7.07 (1.55)	6.46 (1.94)	5.87 (1.67)

Note. Not appl. = Not applicable; DOAF = duration of average fixation, FC = fixation count, VI zone = vehicle impact zone, st. = standardized, non-st. = non-standardized, Info. trans. = information transmission, Subj. performance = subjective performance;
Variables are not winsorized.
